# Osmotic Drug Delivery System as a Part of Modified Release Dosage Form

**DOI:** 10.5402/2012/528079

**Published:** 2012-07-17

**Authors:** Rajesh A. Keraliya, Chirag Patel, Pranav Patel, Vipul Keraliya, Tejal G. Soni, Rajnikant C. Patel, M. M. Patel

**Affiliations:** ^1^Department of Pharmaceutics, Kalol Institute of Pharmacy, Gujarat, Kalol 382721, India; ^2^Department of Pharmaceutics, R. K. College of Pharmacy, Gujarat, Rajkot 360020, India; ^3^Department of Pharmaceutics, DDIT Pharmacy College, Nadiad 387001, India

## Abstract

Conventional drug delivery systems are known to provide an immediate release of drug, in which one can not control the release of the drug and can not maintain effective concentration at the target site for longer time. Controlled drug delivery systems offer spatial control over the drug release. Osmotic pumps are most promising systems for controlled drug delivery. These systems are used for both oral administration and implantation. Osmotic pumps consist of an inner core containing drug and osmogens, coated with a semipermeable membrane. As the core absorbs water, it expands in volume, which pushes the drug solution out through the delivery ports. Osmotic pumps release drug at a rate that is independent of the pH and hydrodynamics of the dissolution medium. The historical development of osmotic systems includes development of the Rose-Nelson pump, the Higuchi-Leeper pumps, the Alzet and Osmet systems, the elementary osmotic pump, and the push-pull system. Recent advances include development of the controlled porosity osmotic pump, and systems based on asymmetric membranes. This paper highlights the principle of osmosis, materials used for fabrication of pumps, types of pumps, advantages, disadvantages, and marketed products of this system.

## 1. Introduction

The pharmaceutical field over the past decade has faced continuing challenges in bringing new drug entity to market. In addition, the cost of developing new drug entity keeps rising and today stands at more than US$ 800 M per new drug entity. Drug delivery research continues to find new therapies for the prevention and treatment of exiting and new diseases. So, a valuable role is played by drug delivery system by providing optimized products for existing drugs in terms of either enhanced or improved presentation of drug to the systemic circulation [1, 2].

Treatment of an acute disease or a chronic illness has been mostly accomplished by delivery of drugs to patients using various pharmaceutical dosage forms. Traditionally, the oral drug delivery has been most widely utilized route of administration among all the routes that have been explored for the systemic delivery of drugs. Conventional oral drug delivery systems are known to provide an immediate release of drug, in which one cannot control the release of the drug and cannot maintain effective concentration at the target site for longer period of time. The oral bioavailability of some drug by conventional drug delivery is very low due to presence of food, in stabilization at pH of the GI tract, degradation by enzymes of GI fluid, change in GI motility, and so forth [3, 4].

Controlled drug delivery systems offer temporal and/or spatial control over the release of drug. Such systems release the drug with constant or variable release rates. Oral controlled drug delivery systems represent the most popular form of controlled drug delivery systems for the obvious advantages of oral route of drug administration. These dosage forms offer many advantages, such as nearly constant drug level at the site of action, prevention of peak-valley fluctuations, reduction in dose of drug, reduced dosage frequency, avoidance of side effects, and improved patient compliance [5, 6].

**Figure 1:**
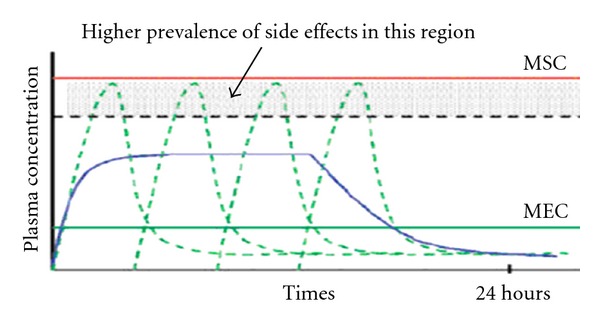
Plasma concentration profile: for conventional dosage form (- - -) and for controlled release dosage form (—).

The oral controlled release system shows a typical pattern of drug release in which the drug concentration is maintained in between the minimum effective concentration (MEC) and maximum safe concentration (MSC) for a prolonged period of time, thereby ensuring sustained therapeutic action (Figure 1).

## 2. Osmotically Controlled Drug Delivery Systems

Osmotic devices are most promising strategy-based systems for controlled drug delivery [7–9]. Osmosis can be defined as the net movement of water across a selectively permeable membrane driven by a difference in osmotic pressure across the membrane. It is driven by a difference in solute concentrations across the membrane that allows passage of water, but rejects most solute molecules or ions. Osmosis is exploited for development of ideal controlled drug delivery system. Osmotic pressure created by osmogen is used as driving force for these systems to release the drug in controlled manner [9].

These systems can be used for both route of administration, that is, oral and implantation. Osmotic pump offers many advantages over other controlled drug delivery systems, that is, they are easy to formulate and simple in operation, improved patient compliance with reduced dosing frequency and more consistence, and prolonged therapeutic effect with uniform blood concentration. Moreover they are inexpensive and their production scaleup is easy [10, 11].

Osmotic drug-delivery systems suitable for oral administration typically consist of a compressed tablet core that is coated with a semipermeable membrane coating. This coating has one or more delivery ports through which a solution or suspension of the drug is released over time. The core consists of a drug formulation that contains an osmotic agent and a water swellable polymer. The rate at which the core absorbs water depends on the osmotic pressure generated by the core components and the permeability of the membrane coating. As the core absorbs water, it expands in volume, which pushes the drug solution or suspension out of the tablet through one or more delivery ports [12, 13].

The key distinguishing feature of osmotic drug delivery systems (compared with other technologies used in controlled-release formulations) is that they release drug at a rate that is independent of the pH and hydrodynamics of the external dissolution medium. The result is a robust dosage form for which the in vivo rate of drug release is comparable to the in vitro rate, producing an excellent in vitro/in vivo correlation. Another key advantage of the present osmotic systems is that they are applicable to drugs with a broad range of aqueous solubilities [14, 15].

The historical development of osmotic systems includes seminal contributions such as the Rose-Nelson pump [16], the Higuchi-Leeper pumps [17], the Alzet and Osmet systems [18], the elementary osmotic pump [19], and the push-pull or GITSR system [20]. Recent advances include the development of the controlled porosity osmotic pump [21, 22], systems based on asymmetric membranes [23–25], and other approaches [12].

## 3. Materials Used in Formulation of Osmotic Pumps

 The following are the materials used in formulation of osmotically regulated system.

**(1) Semipermeable Membrane**

Since the membrane in osmotic systems is semipermeable in nature, any polymer that is permeable to water but impermeable to solute can be selected [26]. Cellulose acetate is a commonly employed semipermeable polymer for the preparation of osmotic pumps. It is available in different acetyl content grades. Particularly, acetyl content of 32% and 38% is widely used. Acetyl content is described by the degree of substitution (DS), that is, the average number of hydroxyl groups on the anhydroglucose unit of the polymer replaced by substituting group. Some of the polymers that can be used for above purpose include cellulose esters such as cellulose acetate, cellulose diacetate, cellulose triacetate, cellulose propionate, cellulose acetate butyrate, and cellulose ethers like ethyl cellulose [27]. Apart from cellulose derivatives, some other polymers such as agar acetate, amylose triacetate, betaglucan acetate, poly(vinyl methyl) ether copolymers, poly(orthoesters), poly acetals and selectively permeable poly(glycolic acid), poly(lactic acid) derivatives, and Eudragits can be used as semipermeable film-forming materials [28]. The permeability is the important criteria for the selection of semipermeable polymers [19].

**(2) Hydrophilic and Hydrophobic Polymers**

These polymers are used in the formulation development of osmotic systems for making drug containing matrix core. The highly water soluble compounds can be coentrapped in hydrophobic matrices and moderately water soluble compounds can be coentrapped in hydrophilic matrices to obtain more controlled release. Generally, mixtures of both hydrophilic and hydrophobic polymers have been used in the development of osmotic pumps of water-soluble drugs [29]. The selection is based on the solubility of the drug as well as the amount and rate of drug to be released from the pump. The polymers are of either swellable or nonswellable nature. Mostly, swellable polymers are used for the pumps containing moderately water-soluble drugs. Since they increase the hydrostatic pressure inside the pump due to their swelling nature, the nonswellable polymers are used in case of highly water-soluble drugs [30]. Ionic hydrogels such as sodium carboxymethyl cellulose are preferably used because of their osmogenic nature. More precise controlled release of drugs can be achieved by incorporating these polymers into the formulations. Hydrophilic polymers such as hydroxy ethyl cellulose, carboxy methylcellulose, hydroxy propyl methylcellulose, high-molecular-weight poly(vinyl pyrrolidone), and hydrophobic polymers such as ethyl cellulose and wax materials can be used for this purpose [31].

**(3) Wicking Agents**

A wicking agent is defined as a material with the ability to draw water into the porous network of a delivery device. The wicking agents are those agents which help to increase the contact surface area of the drug with the incoming aqueous fluid. The use of the wicking agent helps to enhance the rate of drug released from the orifice of the drug. A wicking agent is of either swellable or nonswellable nature [32]. They are characterized by having the ability to undergo physisorption with water. Physisorption is a form of absorption in which the solvent molecules can loosely adhere to surfaces of the wicking agent via Van der Waals interactions between the surface of the wicking agent and the adsorbed molecule. The function of the wicking agent is to carry water to surfaces inside the core of the tablet, thereby creating channels or a network of increased surface area [33]. The examples are colloidal silicon dioxide, PVP and Sodium lauryl sulfate.

**(4) Solubilizing Agents**

For osmotic drug delivery system, highly water-soluble drugs would demonstrate a high release rate that would be of zero order. Thus, many drugs with low intrinsic water solubility are poor candidates for osmotic delivery. However, it is possible to modulate the solubility of drugs within the core. Addition of solubilizing agents into the core tablet dramatically increases the drug solubility [35].

Nonswellable solubilizing agents are classified into three groups,

- Agents that inhibit crystal formation of the drugs or otherwise act by complexation with the drugs (e.g., PVP, poly(ethylene glycol) (PEG 8000) and *β*-cyclodextrin),
- a micelle-forming surfactant with high HLB value, particularly nonionic surfactants (e.g., Tween 20, 60, and 80, polyoxyethylene or poly ethylene containing surfactants and other long-chain anionic surfactants such as SLS),
- citrate esters (e.g., alkyl esters particularly triethyl citrate) and their combinations with anionic surfactants. The combinations of complexing agents such as polyvinyl pyrrolidone (PVP) and poly(ethylene glycol) with anionic surfactants such as SLS are mostly preferred.

**(5) Osmogens**

Osmogens are essential ingredient of the osmotic formulations. Upon penetration of biological fluid into the osmotic pump through semipermeable membrane, osmogens are dissolved in the biological fluid, which creates osmotic pressure buildup inside the pump and pushes medicament outside the pump through delivery orifice. They include inorganic salts and carbohydrates. Mostly, potassium chloride, sodium chloride, and mannitol used as osmogens. Generally combinations of osmogens are used to achieve optimum osmotic pressure inside the system (Table 1) [36].

**(6) Surfactants**

Surfactants are particularly useful when added to wall-forming material. They produce an integral composite that is useful for making the wall of the device operative. The surfactants act by regulating the surface energy of materials to improve their blending into the composite and maintain their integrity in the environment of use during the drug release period. Typical surfactants such as poly oxyethylenated glyceryl recinoleate, polyoxyethylenated castor oil having ethylene oxide, glyceryl laurates, and glycerol (sorbiton oleate, stearate, or laurate) are incorporated into the formulation.

**(7) Coating Solvents**

Solvents suitable for making polymeric solution that is used for manufacturing the wall of the osmotic device include inert inorganic and organic solvents that do not adversely harm the core and other materials. The typical solvents include methylene chloride, acetone, methanol, ethanol, isopropyl alcohol, butyl alcohol, ethyl acetate, cyclohexane, carbon tetrachloride, and water. The mixtures of solvents such as acetone-methanol (80 : 20), acetone-ethanol (80 : 20), acetone-water (90 : 10), methylene chloride-methanol (79 : 21), methylene chloride-methanol-water (75 : 22 : 3) can be used.

**(8) Plasticizers**

In pharmaceutical coatings, plasticizers, or low molecular weight diluents are added to modify the physical properties and improve film-forming characteristics of polymers. Plasticizers can change visco elastic behavior of polymers significantly [37]. Plasticizers can turn a hard and brittle polymer into a softer, more pliable material, and possibly make it more resistant to mechanical stress [9]. Plasticizers lower the temperature of the second order-phase transition of the wall or the elastic modules of the wall and also increase the workability, flexibility, and permeability of the coating solvents. Generally from 0.001 to 50 parts of a plasticizer or a mixture of plasticizers are incorporated into 100 parts of costing materials. PEG-600, PEG-200, triacetin (TA), dibutyl sebacate, ethylene glycol monoacetate, ethylene glycol diacetate, triethyl phosphate, and diethyl tartrate used as plasticizer in formulation of semipermeable membrane [38, 39].

**(9) Pore-Forming Agents**

These agents are particularly used in the pumps developed for poorly water-soluble drugs and in the development of controlled porosity or multiparticulate osmotic pumps [40]. These pore-forming agents cause the formation of microporous membrane. The microporous wall may be formed in situ by a pore-former by its leaching during the operation of the system. The pore-formers can be inorganic or organic and solid or liquid in nature. For example, alkaline metal salts such as sodium chloride, sodium bromide, potassium chloride, potassium sulphate, potassium phosphate, and so forth, alkaline earth metals such as calcium chloride and calcium nitrate, carbohydrates such as sucrose, glucose, fructose, mannose, lactose, sorbitol, and mannitol, and diols and polyols such as poly hydric alcohols, polyethylene glycols, and polyvinyl pyrrolidone can be used as pore-forming agents [41]. Triethyl citrate (TEC) and triacetin (TA) are also used to create pore in the membrane. Membrane permeability to the drug is further increased addition of HPMC or sucrose [42].

## 4. Creation of Delivery Orifice

Osmotic delivery systems contain at least one delivery orifice in the membrane for drug release. The size of delivery orifice must be optimized in order to control the drug release from osmotic systems. On the other hand, size of delivery orifice should not also be too large, otherwise, solute diffusion from the orifice may take place. If the size of delivery orifice is too small, zero-order delivery will be affected because of development of hydrostatic pressure within the core. This hydrostatic pressure may not be relieved because of the small orifice size and may lead to deformation of delivery system, thereby resulting in unpredictable drug delivery. Optimum orifice diameter is in the range of 0.075–0.274 mm. At orifice size of 0.368 mm and above, control over the delivery rate is lost [9].

Delivery orifices in the osmotic systems can be created with the help of a mechanical drill [43]. Laser drilling is one of the most commonly used techniques to create delivery orifice in the osmotic tablet [44]. Laser beam is fired onto the surface of the tablet that absorbs the energy of the beam and gets heated ultimately causing piercing of the wall and, thus forming orifice. It is possible to control the size of the passageway by varying the laser power, firing duration (pulse time), thickness of the wall, and the dimensions of the beam at the wall.

In some of the oral osmotic systems, there is in situ formation of delivery orifice [45]. The system described consists of a incorporation of pore-forming agents into the coating solution. Pore-forming agents are water soluble: upon contact with the aqueous environment, they dissolve in it and leach out from membrane, creatingorifice.

## 5. Types of Osmotic Pumps

### 5.1. Rose-Nelson Pump

Rose and Nelson, the Australian scientists, were initiators of osmotic drug delivery. In 1955, they developed an implantable pump for the delivery of drugs to the sheep and cattle gut [16].

**Figure 2:**
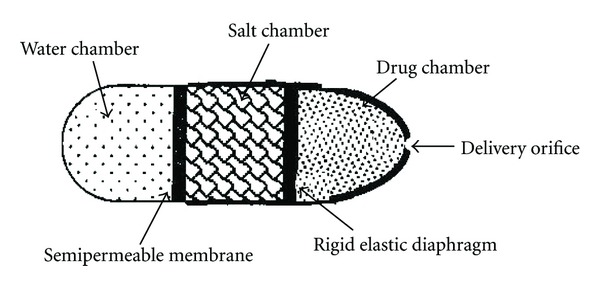
Rose Nelson Pump.

The Rose-Nelson implantable pump shown in Figure 2 is composed of three chambers: a drug chamber, a salt chamber holding solid salt, and a water chamber. A semipermeable membrane separates the salt from water chamber. The movement of water from the water chamber towards salt chamber is influenced by difference in osmotic pressure across the membrane. Conceivably, volume of salt chamber increases due to water flow, which distends the latex diaphragm dividing the salt and drug chambers: eventually, the drug is pumped out of the device.

The kinetics of pumping from Rose Nelson pump is given by the following equation:

(1) dMtdt=(dVdt)·C,

where *dMt/dt* is the drug release rate, *dV/dt* is the volume flow of water into the salt chamber, and *C* represents the concentration of drug in the drug chamber.

(2) dMtdt=AθΔπCl,

where, *A* is the area of semi permeable membrane, Δ*π* is the osmotic pressure gradient, *θ* is the permeability of semipermeable membrane, and l is the thickness of semi permeable membrane.These basic equations are applicable to the osmotically driven controlled drug delivery devices. The saturated salt solution created a high osmotic pressure compared to that pressure required for pumping the suspension of active agent. Therefore, the rate of water entering into the salt chamber remains constant as long as sufficient solid salt is present in die salt chamber to maintain a saturated solution and thereby a constant osmotic pressure driving force is generated.

The major problem associated with Rose-Nelson pumps was that the osmotic action began whenever water came in contact with the semipermeable membrane. This needed pumps to be stored empty and water to be loaded prior to use.

### 5.2. Higuchi-Leeper Osmotic Pump

**Figure 3:**
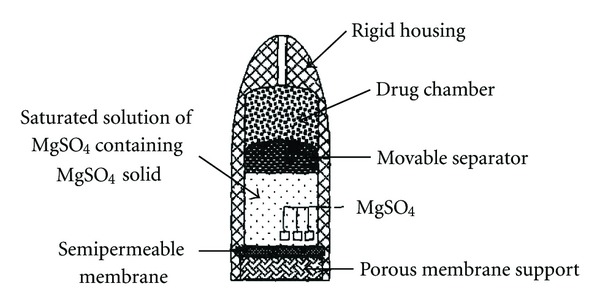
Higuchi-Leeper osmotic pump.

Higuchi and Leeper have proposed a number of variations of the Rose-Nelson pump and these designs have been described in US patents [46, 47], which represent the first series of simplifications of the Rose-Nelson pump made by the Alza Corporation. One of these pumps is illustrated in Figure 3.

The Higuchi-Leeper pump has no water chamber, and the activation of the device occurs after imbibition of the water from the surrounding environment. This variation allows the device to be prepared loaded with drug and can be stored for long prior to use. Higuchi-Leeper pumps contain a rigid housing and a semi permeable membrane supported on a perforated frame; a salt chamber containing a fluid solution with an excess of solid salt is usually present in this type of pump. Upon administration/implantation, surrounding biological fluid penetrates into the device through porous and semipermeable membrane and dissolves the MgSO_4_, creating osmotic pressure inside the device that pushes movable separator toward the drug chamber to remove drug outside the device. It is widely employed for veterinary use. This type of pump is implanted in body of an animal for delivery of antibiotics or growth hormones to animals [17].

**Figure 4:**
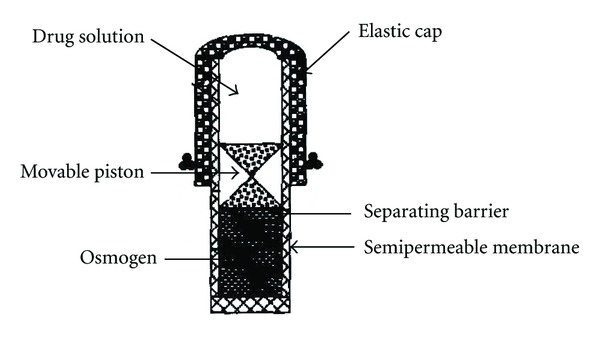
Pulsatile release osmotic pump.

Pulsatile delivery could be achieved by using Higuchi Leeper pump; such modifications are described and illustrated in Figure 4. The Pulsatile release of drug is achieved by drilling the orifice in elastic material that stretches under the osmotic pressure. Pulse release of drug is obtained after attaining a certain critical pressure, which causes the orifice to open. The pressure then reduces to cause orifice closing and the cycle repeats to provide drug delivery in a pulsatile fashion. The orifice should be small enough to be substantially closed when the threshold level of osmotic pressure is not present [49].

### 5.3. Higuchi-Theeuwes Osmotic Pump

**Figure 5:**
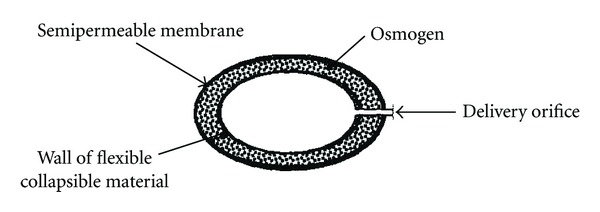
Higuchi Theeuwes Pump.

Higuchi and Theeuwes in early 1970s developed another variant of the Rose-Nelson pump, even simpler than the Higuchi-Leeper pump [50]. This device is illustrated in Figure 5.

 In this device, the rigid housing consisted of a semipermeable membrane. This membrane is strong enough to withstand the pumping pressure developed inside the device due to imbibition of water. The drug is loaded in the device only prior to its application, which extends advantage for storage of the device for longer duration. The release of the drug from the device is governed by the salt used in the salt chamber and the permeability characteristics of the outer membrane [51].

**Figure 6:**
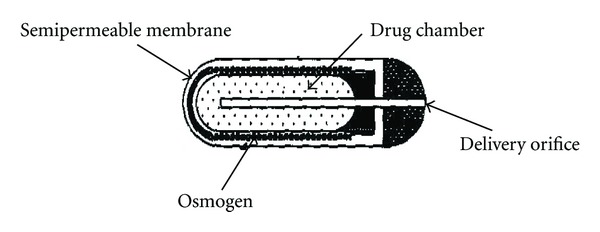
Alzet pump.

 Small osmotic pumps of this form are available under trade name Alzet made by Alza Corporation in 1976. They are used frequently as implantable controlled release delivery systems in experimental studies requiring continuous administration of drugs. Such a implantable Alzet pump is shown in Figure 6.

### 5.4. Elementary Osmotic Pump (EOP)

**Figure 7:**
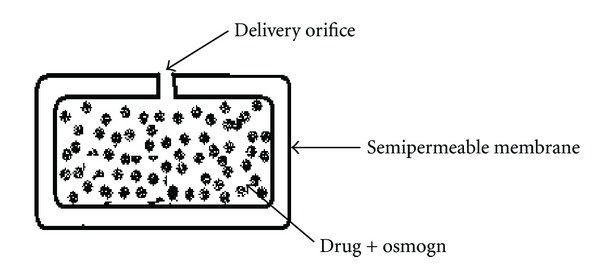
The elementary osmotic pump.

Rose-Nelson pump was further simplified in the form of elementary osmotic pump [20, 52], which made osmotic delivery as a major method of achieving controlled drug release. Elementary osmotic pump shown in Figure 7 was invented by Theeuwes in 1974 and it essentially contains an active agent having a suitable osmotic pressure; it is fabricated as a tablet coated with semi permeable membrane, usually cellulose acetate [32, 53]. A small orifice is drilled through the membrane coating. When this coated tablet is exposed to an aqueous environment, the osmotic pressure of the soluble drug inside the tablet draws water through the semi permeable coating and a saturated aqueous solution of drug is formed inside the device. The membrane is nonextensible and the increase in volume due to imbibition of water raises the hydrostatic pressure inside the tablet, eventually leading to flow of saturated solution of active agent out of the device through a small orifice [19].

The pump initially releases the drug at a rate given by the following equation;

(3) dMtdt=(dVdt)·Cs,

where *dV/dt* depicts the water flow into the tablet and Cs is the solubility of the agent inside the tablet.

### 5.5. Push-Pull Osmotic Pump (PPOP)

**Figure 8:**
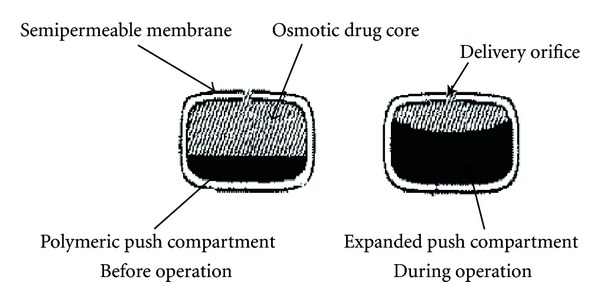
The push-pull osmotic pump (PPOP).

Push-pull osmotic pump is a modification of EOP (Figure 8). Push-pull osmotic pump is delivered both poorly water soluble and highly water soluble drugs at a constant rate. This system resembles a standard bilayer coated tablet. One layer (the upper layer) contains drug in a formulation of polymeric, osmotic agent, and other tablet excipients. This polymeric osmotic agent has the ability to form a suspension of drug in situ. When this tablet later imbibes water, the other layer contains osmotic and colouring agents, polymer and tablet excipients. These layers are formed and bonded together by tablet compression to form a single bilayer core. The tablet core is then coated with semipermeable membrane. After the coating has been applied, a small hole is drilled through the membrane by a laser or mechanical drill on the drug layer side of the tablet. When the system is placed in aqueous environment, water is attracted into the tablet by an osmotic agent in both the layers. The osmotic attraction in the drug layer pulls water into the compartment to form in situ a suspension of drug. The osmotic agent in the nondrug layer simultaneously attracts water into that compartment, causing it to expand volumetrically, and the expansion of nondrug layer pushes the drug suspension out of the delivery orifice [20, 52].

### 5.6. Controlled Porosity Osmotic Pump (CPOP)

**Figure 9:**
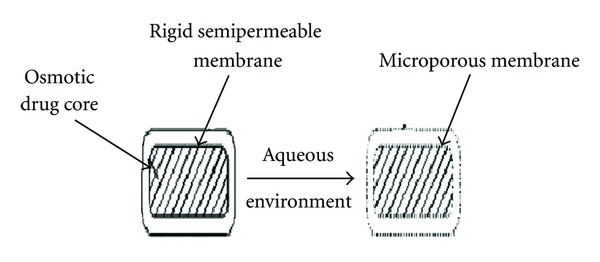
Mechanism of action of controlled porosity osmotic pump.

Figure 9 represents the controlled porosity osmotic pump (CPOP). It is an osmotic tablet wherein the delivery orifices (holes) are formed in situ through leaching of water soluble pore-forming agents incorporated in semipermeable membrane (SPM) (e.g., urea, nicotinamide, sorbitol, etc.). Drug release rate from CPOP depends on various factors like coating thickness, solubility of drug in tablet core, level of leachable pore-forming agent(s) and the osmotic pressure difference across the membrane [54, 55].

There are several obvious advantages inherent to the CPOP system. The stomach irritation problems are considerably reduced, as drug is released from the whole of the device surface rather from a single hole [56]. Further, no complicated laser-drilling unit is required because the holes are formed *in situ*. Scheme describes the drug release phenomenon from a typical CPOP [29].

### 5.7. Liquid-Oral Osmotic (L-OROS) System

Various L-OROS systems available to provide controlled delivery of liquid drug formulations include L-OROS hardcap, L-OROS softcap, and a delayed liquid bolus delivery system. Each of these systems includes a liquid drug layer, an osmotic engine or push layer, and a semipermeable membrane coating. When the system is in contact with the aqueous environment, water permeates across the rate-controlling membrane and activates the osmotic layer (Figure 11) [9, 57].

**Figure 10:**
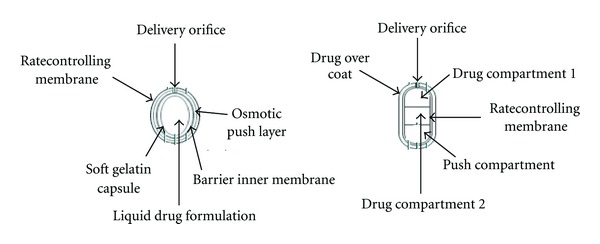
Liquid oral osmotic pump.

**Figure 11:**
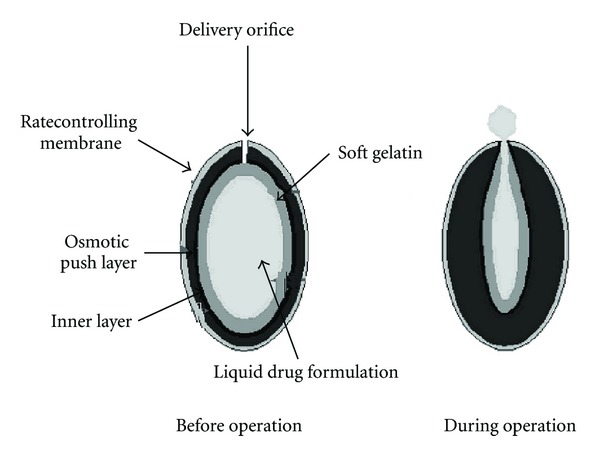
Figure of L-OROS system before and during operation.

 The expansion of the osmotic layer results in the development of hydrostatic pressure inside the system, thereby forcing the liquid formulation to be delivered at the delivery orifice. Whereas L-OROS hardcap and L-OROS softcap systems are designed to provide continuous drug delivery, the L-OROS delayed liquid bolus delivery system is designed to deliver a pulse of liquid drug (Figure 10) [34, 58].

The delayed liquid bolus delivery system comprises three layers: a placebo delay layer, a liquid drug layer, and an osmotic engine, all surrounded by a rate-controlling semipermeable membrane (SPM). The delivery orifice is drilled on the placebo layer end of the capsule shaped device. When the osmotic engine expands, the placebo is released first, delaying release of the drug layer (Figure 10). Drug release can be delayed from 1 to 10 hours, depending on permeability of the rate-controlling membrane and the size of placebo [59].

### 5.8. Sandwiched Osmotic Tablet (SOT)

**Figure 12:**
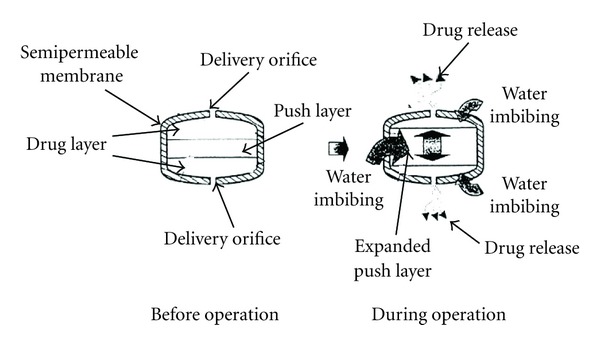
Figure of sandwiched osmotic pump before and during operation.

Figure 12 shows that sandwiched osmotic tablet is composed of polymeric push layer sandwiched between two drug layers with two delivery orifices. When placed in the aqueous environment, the middle push layer containing the swelling agents' swells and the drug is released from the two orifices situated on opposite sides of the tablet; thus sandwiched osmotic tablets (SOTS) can be suitable for drugs prone to cause local irritation of the gastric mucosa (Table 2) [32, 55, 60].

## 6. Conclusion

Osmotic pumps are one of the systems for controlled drug delivery. Osmotic drug delivery systems typically consist of a drug core containing osmogen that is coated with a semipermeable membrane. This coating has one or more delivery ports through which a solution or suspension of the drug is released over time. Various osmotic systems include Rose-Nelson pump, the Higuchi-Leeper pumps, the Alzet and Osmet systems, the elementary osmotic pump, and the push-pull pump. Recent advances include the development of the controlled porosity osmotic pump, L-OROS pump, and sandwiched osmotic tablet. In future, various attempts are made to produce successful osmotic system like pulsatile delivery based on expandable orifice, lipid osmotic pump, telescopic capsule containing mini osmotic pump for delayed release, osmotic bursting osmotic pump, and so forth.

## Figures and Tables

**Table 1:** List of various osmogens with their osmotic pressure [32, 34].

Osmotic pressures of saturated solution of commonly used osmogens	Osmotic pressure (atm)
Sodium chloride	356
Fructose 3	55
Potassium chloride	245
Sucrose	150
Xylitol	104
Sorbitol	84
Dextrose	82
Citric acid	69
Tartaric acid	67
Mannitol	38
Potassium sulphate	39
Lactose	23
Fumaric acid	10
Adipic acid	8
Lactose-fructose	500
Dextrose-fructose	450
Sucrose-fructose	430
Mannitol-fructose	415
Sodium chloride	356
Fructose	335
Lactose-sucrose	250
Potassium chloride	245
Lactose-dextrose	225
Mannitol-dextrose	225
Dextrose-sucrose	190
Mannitol-sucrose	170
Sucrose	150
Mannitol-Lactose	130
Dextrose	82
Potassium sulphate	39
Mannitol	38
Sodium phosphate tribasic·12H_2_O	36
Sodium phosphate dibasic·7 H_2_O	31
Sodium phosphate dibasic·12 H_2_O	31
Sodium phosphate monobasic·H_2_O	28
Sodium phosphate dibasic. Anhydrous	21

**Table 2:** Marketed products of osmotic pump.

Product name	Active pharmaceutical ingredient	Design of osmotic pump
Acutrim	Phenylpropanolamine	Elementary pump osmotic pump [9]
Alpress LP	Prazosin	Push-pull osmotic pump [2]
Cardura XL	Doxazosin	Push-pull osmotic pump [34]
ChronogesicTM	Sufentanil	Implantable osmotic system [8]
Covera HS	Verapamil	Push-pull osmotic pump with time delay [48]
Ditropan XL	Oxybutinin chloride	Push-pull osmotic pump [9]
Dynacirc CR	Isradipine	Push-pull osmotic pump [34]
Efidac 24	Pseudoephiderine	Elementary pump osmotic pump [8]
Efidac 24	Chlorpheniramine meleate	Elementary pump osmotic pump
Glucotrol XL	Glipizide	Push-pull osmotic pump [11]
Invega	Paliperidone	Push-pull osmotic pump [8]
Minipress XL	Prazocine	Elementary osmotic pump [19]
Procadia XL	Nifedipine	Push-pull osmotic pump [48]
Sudafed 24	Pseudoephedrine	Elementary osmotic pump [19]
Viadur	Leuprolide acetate	Implantable osmotic system [9]
Volmex	Albuterol	Elementary osmotic pump [12]
